# Novel Therapeutic/Integrative Approaches for Celiac Disease and Dermatitis Herpetiformis

**DOI:** 10.1155/2012/959061

**Published:** 2012-10-10

**Authors:** Alessio Fasano

**Affiliations:** Mucosal Biology Research and Center for Celiac Research, Health Science Facility II, University of Maryland School of Medicine, Room S345, 20 Penn Street, Baltimore, MD 21201, USA

## Abstract

Celiac disease (CD) is an immune-mediated enteropathy triggered by the ingestion of gluten in genetically susceptible individuals. Gluten is a protein component in wheat and other cereals like rye and barley. At present, the only available treatment is a strict gluten-free diet. Recent advances have increased our understanding of the molecular basis for this disorder. Last decade has seen new scientific developments in this disease and led to the formulation of new concepts of pathophysiology that offer possible targets for new treatments or interventions integrative to the gluten-free diet.

## 1. Introduction 

**Figure 1:**
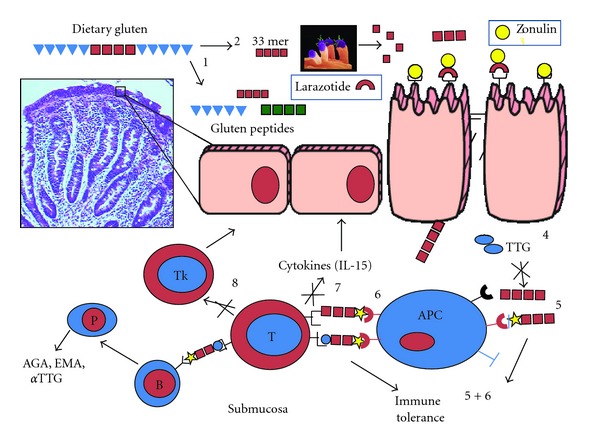
Schematic representation of intestinal mucosal events involved in celiac disease pathogenesis.

Celiac disease (CD) is an immune-mediated chronic enteropathy with a wide range of presenting manifestations of variable severity. It is triggered by the ingestion of gliadin fraction of wheat gluten and similar alcohol-soluble proteins (prolamins) of barley and rye in genetically susceptible subjects with subsequent immune reaction leading to small bowel inflammation and normalization of the villous architecture in response to a gluten-free diet (GFD). CD not only affects the gut, but it is a systemic disease that may cause injury to the skin (dermatitis herpetiformis, the topic of this special issue), liver, joints, brain, heart, and other organs. It is a complex genetic disorder, and human leukocyte antigen (HLA) status appears to be the strongest genetic determinant of risk for celiac autoimmunity. There is a propensity for individuals with CD to carry specific HLA class II alleles, which has been estimated to account for up to 40% of the genetic load [1]. In affected individuals, 95% have either DQ2 (HLA-DQA1 *05-DQB1 *02) or DQ8 (HLADQA1 *03-DQB1 *0302), in comparison with the general population in which 39.5% have either DQ2 or DQ8 [2]. It is the interplay between genes (both HLA and non-HLA associated) and environment (i.e., gluten) that leads to the intestinal damage typical of the disease [3]. Under physiological circumstances, this interplay is prevented by competent intercellular tight junctions (TJs), structures that limit the passage of macromolecules (including gluten) across the intestinal epithelial barrier. Recent evidence suggests that the gluten-induced upregulation of zonulin, a recently described intestinal peptide involved in TJ regulation, is responsible, at least in part, for the aberrant increase in gut permeability characteristic of the early phase of CD [4] and the subsequent abnormal passage of gluten into the lamina propria. Here, the protein is deamidated by tissue transglutaminase and is then recognized by HLA-DQ2/DQ8 bearing, antigen presenting cells, thereby triggering the onset of the CD autoimmune reaction [3] (Figure 1). Similar mechanisms are applied in the autoimmune response to gluten targeting the skin leading to dermatitis herpetiformis (DH). Given the undisputable role of gluten in causing inflammation and immune-mediated tissue damage, CD and DH represent unique models of autoimmunity in which, in contrast to most other autoimmune diseases, a close genetic association with HLA genes (DQ2 and/or DQ8), a highly specific humoral autoimmune response (autoantibodies to tissue transglutaminase), and, most importantly, the triggering environmental factor (gluten), are known. This information provides the rationale for the treatment of the disease based on complete avoidance of gluten-containing grains from the patients' diet, a task complicated by several factors, including poor compliance, inaccurate information, low level of awareness among health care providers, a food labeling policy still in progress, and the lack of consensus on proper safe gluten thresholds.

## 2. The Rationale for Alternative/Integrative Approaches to a Gluten-Free Diet 

The cornerstone of treatment of CD and DH is a lifelong adherence to a strict GFD in which proteins from wheat, rye, barley, and related cereals are eliminated from the diet. Gluten is, however, a common (and in many countries unlabeled) ingredient in the human diet, presenting a big challenge for CD patients. Gluten-free products are not widely available and are more expensive than their gluten-containing counterparts. Dietary compliance is therefore suboptimal in a large proportion of patients. More than 50% of subjects that embrace a diet for medical reasons (hypertension, obesity, high cholesterol, diabetes, renal failure, etc.) fail to comply over time [5], making any diet therapy a high-risk proposition. Furthermore, even when compliance is not an issue, a high percentage of CD subjects on a GFD that are symptom free and test negative to CD serology show persistence of sever intestinal damage [6, 7]. Therefore, treatments alternative to the GFD or integrative to the diet in order to minimize cross-contamination accidents typically occurring outside patients' households would represent desirable interventions to minimize the risk of complications associated to prolonged gluten exposure in subjects affected by CD and DH.

## 3. Gluten Contamination: How Much Is Too Much? 

A GFD completely devoid of gluten is unrealistic. CD and DH patients are exposed to products containing trace amounts of gluten, even when the products are sold as naturally gluten free. In order to estimate the safe threshold for daily gluten intake, the amount of residual gluten in gluten-free products and the total intake of these products must be considered. Provided that we can demonstrate that the use of a variety of gluten-free products results in both clinical and histological recovery, we can assume that the gluten level in these products is acceptable. Most wheat starch-based gluten-free products contain trace amounts of gluten [8]. These products were verified to be safe in clinical practice in a prospective controlled study where no differences in histology, serology, or quality of life were seen between wheat starch-based and naturally gluten-free products [9]. There is little information in the literature on minimal disease-eliciting doses of gluten for sensitive individuals [10]. Literature review suggests that an upper limit for gluten content in food, which would be safe for sufferers from CD, should lie between 10 and 100 mg daily intake [10]. A more evidence-based definition of this limit was identified recently by a recent study that evaluated the effects of exposure to either 10 or 50 mg of purified gluten per day for 3 months with a population of 49 celiac disease individuals in a double-blind, placebo-controlled trial [6]. The results suggest that minimal mucosal abnormalities occur even following a strict GFD, that both 10 mg and 50 mg daily gluten are clinically well tolerated, but that there is a trend for mucosal changes to occur at the 50 mg dose [6]. There is therefore an urgent need to develop safe and effective therapeutic alternatives to the GFD, keeping in mind that any of these alternative approaches needs to match the high level of safety of the diet therapy.

## 4. Why Gluten Is Harmful to CD and DH Subjects 

In order to identify possible targets for therapies alternative to a GFD, it would be helpful to review the significant progress made during the past decade on CD pathogenesis. CD is now considered to be a T-cell-mediated, chronic inflammatory disorder with an autoimmune component. Altered processing by intraluminal enzymes, changes in intestinal permeability, and activation of innate immunity mechanisms seem to precede the activation of the adaptive immune response [11]. In recent years, much has been discovered about the genetic and immunologic aspects of CD [4]. However, little is known about the possible interactions of gliadin (and/or its peptide derivatives) with intestinal epithelia and the mechanism(s) through which it crosses the epithelial barrier to reach the submucosa. Under physiological circumstances, intestinal epithelia are almost impermeable to macromolecules such as gliadin [12]. In CD, paracellular permeability is enhanced, and the integrity of TJ system is compromised [13–15]. The upregulation of zonulin, a recently described intestinal peptide involved in TJ regulation [16, 17], appears to be responsible, at least in part, for the increased gut permeability characteristic of CD [18]. Further, persistent presence of inflammatory mediators such as TNF-*α* and interferon (IFN)-*γ* has been shown to increase the permeability across the endothelial and epithelial layers [19, 20], suggesting that the initial breach of the intestinal barrier function caused by zonulin can be perpetuated by the inflammatory process after the access of gliadin to the submucosa [21]. Additionally, evidence exists suggesting also a transcellular passage of gliadin, particularly when the mucosal damage is already established and the transferrin receptor (CD71) that mediates this transport is expressed on the luminal side of enterocytes, so promoting retrotransport of secretory immunoglobulin (SIg) A-gliadin complexes [22, 23]. Direct evidence of the rapid activation of the innate immune system has been proven in organ culture studies. Interestingly, most of these events of innate immune activation were inhibited by antibodies neutralizing interleukin (IL)-15 [10], thus confirming the key role of this cytokine as a mediator of intestinal mucosal damage induced by ingestion of gliadin. The activation of lamina propria T cells by gliadin peptides in the context of HLA-DQ2 or DQ8 molecules has long been recognized as one of the key events in the pathogenesis of CD. A large number of T-cell-stimulating peptides have been characterized in gluten proteins [24–26]. Studies by Arentz-Hansen et al. [24] and Vader et al. [25, 26] have provided a model to explain the interplay between gliadin, DQ2 or DQ8 and tTG. In fact, these gliadin-specific T-cell responses have been found to be enhanced by the action of tTG. tTG converts glutamine residues into glutamic acid, which results in higher affinity of these gliadin peptides for HLA-DQ2 or HLADQ8. Recent studies have identified in the sequence motifs QXP, the glutamine residues that are preferentially substrate for tTG-mediated deamidation [26]. The repertoire of gluten peptides involved in the disease pathogenesis is greater than appreciated previously and may differ between children and adult patients [24]. Although there are at least 50 T-cell-stimulatory epitopes in gluten proteins, a unique 33 mer gliadin fragment is the most immunogenic peptide because it harbors six overlapping epitopes. Moreover, it is resistant to the enzymatic degradation by gastric acidity and pancreatic and brush border peptidases. This peptide might reach the immune districts of intestinal mucosa in an intact and stimulatory form [27]. Furthermore, the 33 mer peptide does not require further processing in antigen presenting cells for T-cell stimulation because it binds to DQ2 molecules with a pH profile that promotes extracellular binding [28]. The pattern of cytokines produced after gliadin activation is clearly dominated by IFN-*γ* (Th1 skewed) [29]. IFN-*γ*-dependent signaling pathways have been found to be enhanced in CD. 

## 5. Potential New Therapies: Prevention versus Treatment 

**Figure 2:**
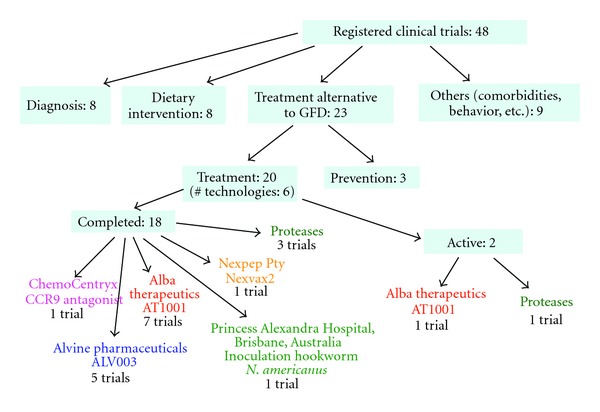
Current clinical trials in celiac disease involving preventive and therapeutic interventions. Data obtained from http://www.clinicaltrials.gov/ update to July 10th 2012.

The aforementioned progress made in the understanding of the cellular and molecular basis of CD led to the identification of potential targets for preventive or therapeutic interventions [30, 31] (Figure 2). 

### 5.1. Prevention

Several retrospective studies have suggested that the time of gluten introduction in the diet of infants at risk for CD may affect the incidence of the disease. However, the data supporting this hypothesis are circumstantial, limited by their retrospective design, and often criticized by alternative interpretations suggesting that the delay in gluten exposure merely postpones the onset of symptoms rather than preventing the disease. In order to clarify the role of infant nutrition on the risk of CD development in at-risk infants, two large prospective, intervention studies that are currently active have been recently initiated [30]. The results of these long-term studies will be available within the next few years. The PreventCD Family Study is currently ongoing in 10 European countries and a total of 1,000 children are involved. The participating children and mothers are to be followed for a period of 1–3 years. The general hypothesis of this study is that small amounts of gluten are administered gradually to induce oral immune tolerance to gluten. The second large study is a multicenter study from Italy aimed at evaluating the role of age at gluten introduction on CD-related autoimmune serological changes in a large cohort of at-risk infants (first-degree relatives of patients with CD). So far more than 700 infants have been enrolled and preliminary data suggest that postponing gluten introduction in the infant's diet at age 12 months decreases the prevalence of CD (Catassi C, personal communication). Both studies necessitate of much longer follow-up analysis to establish whether timing of gluten exposure can really prevent CD or merely delay its onset.

### 5.2. Treatment with Enzyme Therapy

It has been shown that because of the high-proline content, gliadin peptides are highly resistant to digestive processing by pancreatic and brush border proteases [27]. Enzyme supplement therapy with the use of bacterial prolyl endopeptidases has been proposed to promote complete digestion of cereal proteins and thus destroy T-cell multipotent epitopes. One of these enzyme formulations, called ALV003, is currently in clinical trials and has shown promising safety and efficacy data. ALV003 has been administered both in the fasted state and with a gluten-containing meal. All ALV003 doses were well tolerated, and no serious adverse events or allergic reactions were observed. Gastric aspirates collected 30 min following a meal showed that 100 and 300 mg ALV003 degraded 75 ± 10% in the fasting phase and 88 ± 5% in the meal phase of one gram of wheat bread gluten [32]. It remains to be assessed whether the residual amount of undigested gluten can cause arm in the long term. An alternative approach to reduce gluten toxicity is based on a pretreatment of whole gluten or gluten-containing food with bacterial-derived peptidase [33]. Enzymatic detoxification of gluten has the potential to be an effective method for producing more palatable gluten-free products and possibly treating CD. Proteases of certain lactobacilli present in sourdough are able to proteolyze proline-rich gluten peptides [33]. CD patients subjected to an acute challenge tolerated breads produced with sourdough (lactobacillus digested) better than those with baker's yeast [33]. 

## 6. Engineered Grains and Inhibitory Gliadin Peptides

Breeding programs and/or transgenic technology may lead to production of wheat that is devoid of biologically active peptide sequences. Site-directed mutagenesis of wheat, which would not affect the baking properties, has also been proposed, although the number and the repetition of such sequences in wheat render this approach difficult. The identification of specific epitopes may also provide a target for immunomodulation of antigenic peptides. According to the nature of amino acid residue in the position interacting with the specific TCR, peptide recognition can turn out in a cellular activation (agonist), ignorance (null peptides), or unresponsiveness, known also as anergy (antagonist). Peptide analogues of gliadin epitope(s) can be engineered with antagonistic effects of native peptide(s). Of course, the chances of success of using analogue peptides to modulate specific immune responses could be hampered by the great heterogeneity of gliadin T-cell epitopes so far identified. Further studies aimed to elucidate the hierarchy of pathogenic gliadin epitopes, and their core region would be of crucial importance for engineering peptide-based therapy. 

## 7. Immunomodulatory Strategies 

The autoantigenic enzyme tissue transglutaminase (TTG) is mainly expressed in the lamina propria, and its expression is upregulated by various stimuli, such as mechanical stress or bacterial/viral infection, during active CD. The enzyme catalyzes transamidation between a glutamine residue of a glutamine-donor protein and a lysine residue of a glutamine-acceptor protein, linking these proteins with a stable intermolecular isopeptide bond and increasing their rate of phagocytosis by antigen-presenting cells [34]. Although the precise molecular details of this interaction in vivo remain unclear, selective inhibition of TTG in the small intestine might represent a therapeutically useful strategy for countering the immunotoxic response to dietary gluten in CD. The substitution of a glutamine residue with 6-diazo-5-oxo-norleucine (DON) transforms an immunodominant gluten peptide into a potent inhibitor of tissue transglutaminase [35]. DON-modified peptides could be useful for the study and therapy of CD. The efficacy and side effects of TTG inhibitors as a treatment of CD are unknown. The crucial role of the HLA in CD development makes it an obvious target for therapeutic intervention. Blocking of peptide presentation by DQ2 is an attractive approach for a new treatment of CD because DQ2 (or DQ8) is a necessary but insufficient genetic component for disease development. Furthermore, other immunomodulatory targets, including IL-10, are possible alternative tools for promoting tolerance. However, evidence that gluten toxicity is not dependent only on T-cell recognition is growing. In this regard, the mechanism of toxicity of peptide remains unknown. Activation of innate immunity has been demonstrated, and antibodies to IL-15 have been proposed, particularly in the treatment of refractory sprue because of the IEL-activating role of IL-15 [10]. Nevertheless, one should realize that treated CD is a benign condition and dietary treatment is safe, although strenuous. Therefore, any immunomodulatory approach must have a safety profile equivalent to that of the GFD but with the advantage of increased compliance.

## 8. Correction of the Intestinal Barrier Defect 

The primary functions of the gastrointestinal tract have traditionally been perceived to be limited to the digestion and absorption of nutrients and electrolytes and to water homeostasis. A more attentive analysis of the anatomic and functional arrangement of the gastrointestinal tract, however, suggests that its barrier function and ability to regulate the trafficking of macromolecules between the environment and the host are other extremely important functions of this organ. Together with the gut-associated lymphoid tissue and the neuroendocrine network, the intestinal epithelial barrier, with its intercellular tight junctions, controls the equilibrium between tolerance and immunity to non-self antigens. When the finely tuned trafficking of macromolecules is dysregulated in genetically susceptible individuals, both intestinal and extraintestinal autoimmune disorders can occur [36]. This new paradigm subverts traditional theories underlying the development of autoimmunity, which are based on molecular mimicry and/or the bystander effect, and suggests that the autoimmune process can be arrested if the interplay between genes and environmental triggers is prevented by reestablishing the intestinal barrier function. A common denominator of autoimmune diseases is the presence of several preexisting conditions that lead to an autoimmune process. The first is the genetic susceptibility of the host immune system to recognize, and potentially misinterpret, an environmental antigen presented within the gastrointestinal tract. Second, the host must be exposed to the antigen. Finally, the antigen must be presented to the gastrointestinal mucosal immune system following its paracellular passage from the intestinal lumen to the gut submucosa, which is normally prevented by competent tight junctions [30]. In many cases, increased intestinal permeability seems to precede disease and causes an abnormality in antigen delivery that triggers the multiorgan process leading to the autoimmune response [36].

Taking the information above into consideration, it is conceivable to propose that the pathogenesis of autoimmune diseases, including CD, can now be described by the following three key points. First, autoimmune diseases involve a miscommunication between innate and adaptive immunity. Second, molecular mimicry or bystander effects alone might not explain entirely the complex events involved in the pathogenesis of autoimmune diseases. Rather, the continuous stimulation by non-self antigens (environmental triggers) seems to be necessary to perpetuate the process. Contrary to general belief, this concept implies that the autoimmune response can theoretically be stopped and perhaps reversed if the interplay between genes predisposing individuals to the development of autoimmunity and environmental triggers is prevented or eliminated. Third, in addition to genetic predisposition and exposure to triggering non-self antigens, the loss of the protective function of mucosal barriers that interface with the environment (mainly the gastrointestinal and lung mucosa) is necessary for autoimmunity to develop. Based on this theory, it is possible to conceptualize that the removal of any of the three elements necessary to develop autoimmunity (i.e., genetic predisposition, exposure to the environmental trigger(s), or defect of the intestinal barrier function) would be a valid therapeutic option. Given that elimination of the predisposing genes is not a valuable option and that the removal of the trigger antigen (an option available only for CD) has its own challenges (see above), the correction of the intestinal barrier defects may represent an innovative therapeutic alternative. Small intestinal permeability abnormalities are seen in untreated CD patients, which return to normal on a GFD [37]. The use of the zonulin inhibitor AT1001 to correct intestinal barrier defects has been already successfully explored in an animal model of autoimmunity [38]. More recently, AT1001 (now called larazotide acetate) has been tested in an inpatient, double-blind, randomized placebo-controlled human clinical trial to determine its safety, tolerability, and preliminary efficacy [39]. No increase in adverse events was recorded among patients exposed to larazotide acetate as compared to placebo. Following acute gluten exposure, a 70% increase in intestinal permeability was detected in the placebo group, while no changes were seen in the Larazotide acetate group [39]. After gluten exposure, IFN-*γ* levels increased in 4 out of 7 patients (57.1%) of the placebo-group, but only in 4 out of 14 patients (28.6%) of the larazotide group. Gastrointestinal symptoms were significantly more frequent among patients of the placebo group as compared to the larazotide acetate group [39]. Combined, these data suggest that larazotide is well tolerated and appears to reduce gluten-induced intestinal barrier dysfunction, proinflammatory cytokine production, and gastrointestinal symptoms in celiac patients. While the effect of larazotide on assembly and regulation of intercellular TJ and subsequent mucosal inflammation has been amply studied [40, 41], its possible impact on transcellular gluten trafficking remains to be established. 

## 9. Summary 

Although GFD is considered the only effective treatment for individuals with CD and DH, it has been recognized that its implementation is challenging and most of the time suboptimal. A better understanding of the complexity of the genetic/environmental interaction responsible for CD and DH development opens the way to explore alternative therapeutic strategies [42]. It is well possible that reducing the “strength” or the access of the environmental component will prevent disease recurrence, particularly in those patients with a lower genetic load of predisposing genes.
